# Composites Derived from Aluminium-Modified Biphasic Calcium-Phosphate for Bone Regeneration

**DOI:** 10.3390/biomimetics10120824

**Published:** 2025-12-09

**Authors:** Raluca Lucacel-Ciceo, Roxana Dudric, Razvan Hirian, Iulia Lupan, Oana Koblicska, Roxana Strimbu, Radu George Hategan, Dorina Simedru, Zorita Diaconeasa

**Affiliations:** 1 Faculty of Physics, Babes-Bolyai University, 1 M. Kogalniceanu St., 400084 Cluj-Napoca, Romania; razvan.hirian@ubbcluj.ro (R.H.); roxana.strimbu@stud.ubbcluj.ro (R.S.); radu.hategan@ubbcluj.ro (R.G.H.); 2 Institute for Interdisciplinary Research in Bio-Nano-Sciences, Babes-Bolyai University, 42 Treboniu Laurian St., 400271 Cluj-Napoca, Romania; iulia.lupan@ubbcluj.ro (I.L.); oana.koblicska@ubbcluj.ro (O.K.); 3 Faculty of Biology and Geology, Babes-Bolyai University, 44 Republicii St., 400015 Cluj-Napoca, Romania; 4 INCDO-INOE 2000, Subsidiary Research Institute for Analytical Instrumentation, 67 Donath St., 400293 Cluj-Napoca, Romania; dorina.simetru@icia.ro; 5 Department of Chemistry, Biochemistry and Molecular Biology, University of Agricultural Sciences and Veterinary Medicine, 3-5 Calea Manastur St., 400372 Cluj-Napoca, Romania; zorita.sconta@usamvcluj.ro

**Keywords:** biphasic calcium phosphates, β-TCP, hydroxyapatite, aluminium, collagen, acetylsalicylic acid, cell viability, antimicrobial activity, bone regeneration

## Abstract

In this research, aluminium-doped biphasic calcium phosphate (Al-BCP) was synthesized by co-precipitation and formulated with hydrolyzed collagen and acetylsalicylic acid (ASA) to yield composites designed as a new class of bone-regenerative biomaterials with enhanced biological performance. Undoped and Al-modified powders (5/10 wt% Al precursor) were prepared at 40 °C (pH ~ 11) and calcined at 700 °C, and composites were produced at a 1:1:0.1 mass ratio (ceramic–collagen–ASA). Structure and chemistry were assessed by X-ray diffraction (XRD), Fourier-transform infrared (FTIR) and Raman spectroscopies, and X-ray photoelectron spectroscopy (XPS). Morphology and elemental distribution were examined by scanning electron microscopy/energy-dispersive X-ray spectroscopy (SEM/EDX). Biological performance was preliminarily evaluated using HaCaT (immortalized human keratinocytes) viability and antibacterial assays against *Staphylococcus aureus* and *Escherichia coli*. XRD confirmed a biphasic hydroxyapatite/β-tricalcium phosphate system and showed that Al incorporation shifted the phase balance toward hydroxyapatite (HAp fraction 54.8% in BCP vs. ~68.6–68.7% in Al-doped samples). FTIR/Raman preserved BCP vibrational signatures and revealed collagen/ASA bands in the composites. XPS/EDX verified the expected composition, including surface N 1s from organics and Al at ~2–5 at% for doped samples, with surface Ca/P ≈ 1.15–1.16. SEM revealed multigranular microstructures with homogeneous Al distribution. All composites were non-cytotoxic (≥70% viability); M_Al10_Col_ASA exceeded 90% viability at 12.5% dilution. Preliminary antibacterial assays against Gram-positive and Gram-negative strains showed modest, time-dependent reductions in CFU relative to controls. These results corroborate the compositional/structural profile and preliminary biological performance of Al-BCP–collagen–ASA composites as multifunctional bone tissue engineering materials that foster a bone-friendly microenvironment, warranting further evaluation for bone regeneration.

## 1. Introduction

Bone defects arising from trauma, infection, tumour resection, or revision arthroplasty remain a major clinical challenge, motivating the development of bioactive materials that couple osteoconduction with infection control and immunomodulation [1,2,3,4,5,6]. Calcium–phosphate (CaP) ceramics are widely used owing to their chemical similarity to bone mineral and their favourable integration profiles. Among these, biphasic calcium phosphate (BCP)—a mixture of hydroxyapatite (HAp-Ca_10_(PO_4_)_6_(OH)_2_) and β-tricalcium phosphate (β-TCP-Ca_3_(PO_4_)_2_)—offers a very good balance between stability and resorption, enabling controlled remodelling of the grafted site [7,8,9]. Both HAp and β-TCP are biocompatible, nontoxic, resorbable, and non-inflammatory, and they cause neither immune nor irritating responses and have excellent osteoconductive abilities [10,11,12,13,14]. HAp provides long-term structural persistence, whereas β-TCP resorbs more rapidly (up to 10–20 times faster than HAp), releasing Ca^2+^ and P^+^ ions that can stimulate osteogenesis and angiogenesis when appropriately dosed [15,16,17,18]. Recent studies further show that tuning BCP crystallinity and surface structure can improve osteogenic outcomes in vitro and in vivo, and collagen–CaP constructs better mimic bone’s hierarchical matrix and enhance cell response [19,20,21].

Aluminum in the form of alumina (Al_2_O_3_) is widely regarded as a bioinert structural ceramic for load-bearing orthopedics and dental applications, owing to its chemical stability, corrosion resistance, and excellent wear behaviour; dense alumina bearings have a long clinical track record in total hip arthroplasty and remain a reference material in modern ceramic prostheses [22]. In parallel, aluminum incorporation in hydroxyapatite (HAp), typically as Al^3+^ substitution at Ca sites or as surface-associated Al–phosphate environments, modulates HAp’s lattice and surface chemistry (unit-cell contraction, crystallite size/morphology shifts), which can translate into tuneable mechanical/biological performance of HAp powders, coatings, and composites [23]. Studies on Al-doped HAp report altered lattice parameters with controlled nano crystallinity and preserved biocompatibility, supporting the broader view that cation substitution (including Al^3+^) is a viable route to optimize HAp’s mechanical strength, dissolution, and interfacial response for bone repair [24,25,26].

Collagen is the natural organic counterpart of bone mineral, and when combined with calcium phosphates (e.g., hydroxyapatite/β-TCP), it yields biomimetic composites that embraces bone’s hierarchical structure, enhancing cell adhesion, osteoconduction, and matrix deposition. Bone itself is a mineralized collagen nanocomposite; emulating this architecture with collagen–apatite scaffolds improve biological performance and load transfer at relevant length scales [27]. Chemically co-precipitated or self-organized HAp–collagen constructs reproduce bone-like nano assembly and show favourable mechanical and biological responses in vitro and in vivo, including integration and replacement by new bone [28]. Across processing routes (foams, hydrogels, 3D-printed inks), mineralized collagen matrices support osteoblast attachment/proliferation and guide new bone formation in defect models, reflecting synergistic cues from the collagen fibrils and the calcium-phosphate phase [29]. Reviews and experimental studies consistently report that adding nanocrystalline apatite to collagen enhances osteogenic readouts and osteointegration compared with collagen or apatite alone, while retaining porosity and resorption characteristics important for remodelling [30]. In preclinical settings, collagen–CaP composites (including brushite/β-TCP or HAp variants) improve early cell adhesion and bone fill, underscoring collagen’s role as a bioactive matrix that complements the osteoconductive mineral [31].

Incorporating acetylsalicylic acid (ASA, aspirin) into calcium-phosphate or hybrid bioceramics is a rational strategy to improve early healing because ASA inhibits cyclo-oxygenase enzymes (COX) and suppresses prostaglandin E_2_ (key drivers of post-surgical inflammation in bone) thereby dampening the inflammatory microenvironment without sacrificing cytocompatibility; in scaffold formats, controlled/sustained release of ASA has been shown to reduce inflammatory markers and enhance osteogenic responses in vitro and in vivo [32]. Beyond inflammation control, ASA (and its metabolite salicylic acid) can modulate bacterial growth and biofilms, offer adjunct anti-infective potential when is co-delivered with a bone graft—though effects are strain- and dose-dependent and can be pro-biofilm for *S. aureus* under some conditions, underscoring the need to tune dose and release [33]. In a recent study, optimized hydrogels integrating PVA/gelatin/sodium alginate with aspirin (ASA) and nano-hydroxyapatite (nHAp) improved MC3T3-E1 viability, promoted osteogenic differentiation, and modulated the inflammatory microenvironment, underscoring the value of coupling CaP with ASA [34]. Finally, ASA-releasing CaP/aerogel or hydrogel-HAp scaffolds have demonstrated enhanced bone formation alongside anti-inflammatory activity, supporting ASA as a useful bioactive in next-generation bioceramic systems [35].

Despite strong evidence for collagen–CaP osteoconduction, growing interest in ASA-releasing bone scaffolds for early immunomodulation/antimicrobial support, and demonstrated feasibility of Al-doped apatite’s [23,24,25,26], we found no prior report combining Al-doped BCP with collagen and ASA into a single composite intended to unify phase-tuned osteoconduction with controlled anti-inflammatory/antibacterial functionality. The closest analogues are collagen–HAp or BCP–collagen matrices without ASA [27,28,29,30,31], and ASA-loaded hydrogels/aerogels or nano HAp systems without Al-doped BCP [34,35]. This identifies a specific materials gap at the intersection of phase engineered BCP, biomimetic collagen, and ASA delivery.

Recognizing that biphasic calcium phosphate (BCP) is a widely adopted, clinically used bone substitute owing to its osteoconductivity and safe resorption, yet still limited by early postoperative inflammation and infection risk, this work aimed to synthesize Al-doped BCP and elucidates its structure by XRD, FTIR, Raman, XPS spectroscopy, and SEM/EDX and fabricate BCP–collagen–aspirin composites, to evaluate their structural features and in-vitro preliminary biological performance (cytocompatibility and antibacterial activity), addressing the need for next-generation calcium phosphate systems that combine osteoconduction process with immunomodulatory and anti-infective functions.

## 2. Materials and Methods

### 2.1. Materials

Analytical-grade reagents used include calcium nitrate tetrahydrate (Ca(NO_3_)_2_·4H_2_O, ≥99.0%, CAS 13477-34-4; Merck Millipore, Darmstadt, Germany,), diammonium hydrogen phosphate ((NH_4_)_2_HPO_4_, ≥99.0%, CAS 7783-28-0; Merck Millipore), aluminium nitrate nonahydrate (Al(NO_3_)_3_·9H_2_O, ≥98.5%, CAS 7784-27-2; Merck Millipore), citric acid (≥99.5%, CAS 5949-29-1; Chempur, Poland), and ammonium hydroxide solution (25% p.a., CAS 1336-21-6; Chempur, Poland). Double-distilled water was used throughout.

### 2.2. Synthesis of BCP and Al-Doped BCP Powders

A 0.9 M Ca(NO_3_)_2_·4H_2_O solution was prepared by dissolving 10.63 g in 50 mL water; a 0.6 M (NH_4_)_2_HPO_4_ solution was prepared by dissolving 3.96 g in 50 mL water. Both solutions were magnetic stirred at 400 rpm and maintained at 40 °C. Citric acid (3 wt% relative to the calcium solution) was added as dispersant to the Ca(NO_3_)_2_·4H_2_O solution. Under magnetic stirring (400 rpm) at 40 °C, the phosphate solution was added dropwise to the calcium/citric solution, and the resulting mixture was stirred continuously for a total of 2 h. The pH was adjusted to ~11 with ammonium hydroxide. The resulting suspensions were cooled to room temperature, aged 24 h, filtered, and washed three times with water. Powders were dried at 80 °C for 24 h (drying oven, BIOBASE, Jinan, China) and subsequently calcined at 700 °C for 1 h at a heating rate of 5 °C min^−1^ (Caloris Microtherm furnace) to obtain biphasic calcium phosphate (BCP).

Al-modified powders were prepared identically, except that Al(NO_3_)_3_·9H_2_O was added to the calcium solution at 5 or 10 wt% relative to the combined mass of Ca(NO_3_)_2_·4H_2_O and (NH_4_)_2_HPO_4_ precursors (0.73 g and 1.46 g, respectively). Samples are denoted M (BCP), M_Al5 (BCP + 5 wt% Al precursor), and M_Al10 (BCP + 10 wt% Al precursor).

### 2.3. Preparation of BCP/Collagen/ASA Composites

Composite samples were prepared by mixing predetermined amounts of ceramic powders (M, M_Al5, and M_Al10) with hydrolyzed marine collagen (Swedish Collagen) and acetylsalicylic acid (ASA; TCI Chemicals, Tokyo, Japan) at a mass ratio of 1:1:0.1 (ceramic–COL–ASA, wt/wt/wt). Mixtures were mechanically blended (mortar and pestle), ultrasonicated at 120 W for 5 min, and then mixed in a 3D tumbler mixer for 30 min to ensure homogeneity. The resulting composites are hereafter denoted as M_Col_ASA, M_Al5_Col_ASA, and M_Al10_Col_ASA.

### 2.4. Characterization

The X-ray diffraction (XRD) data were recorded at room temperature using a Bruker D8 Advance diffractometer with Cu-K_α_ radiation (λ = 1.5405 Å). Data were acquired in the 2θ range of 10–60° with a step size of 0.03°. FullProf Suite software [36] was used for multiphase Rietveld refinement of the powder diffraction data [37].

The FTIR absorption spectra of the samples were recorded with a JASCO FTIR 6200 spectrometer, in the range 400–4000 cm^−1^, at room temperature, spectral resolution of 4 cm^−1^, using the KBr pellet technique.

For the Raman analysis, a confocal Renishaw^®^InVia microscope (Renishaw plc, Wotton-under-Edge, Gloucestershire, UK) equipped with a frequency-doubled Nd:YAG laser operating at 532 nm was employed. The Raman spectra were recorded in the range of 0–1400 cm^−1^ with an acquisition time of 40 s (10 s integration time and four accumulations) using a laser power (measured at the sample surface) of approximatively 113 mW, while the spectral resolution of the spectrometer was ~2 cm^−1^. Before measuring the samples, a calibration procedure using an internal silicon reference was performed. For the baseline correction, the Wire 4.2 software provided by Renishaw plc (Wotton-under-Edge, Gloucestershire, UK), with the InVia spectrometer was used.

The FTIR and Raman spectra were processed by using OriginPro 2019 software (OriginLab, Nothampton, MA, USA).

X-ray photoelectron spectroscopy (XPS) measurements were conducted on finely powdered samples using a SPECS PHOIBOS 150 MCD system equipped with monochromatic Al Kα source (250 W, *hν* = 1486.6 eV), hemispherical analyzer and multichannel detector, under ultra-high-vacuum conditions (the pressure in the analysis chamber was in the range of 10^−8^–10^−9^ mbar). Surface charging during the XPS analysis was avoided by using an electron gun. All spectra were calibrated against the C1s photoelectron peak at 284.8 eV. The elemental composition on samples surface was determined from survey spectra acquired in the binding energy range of 0–1200 eV at pass energy of 60 eV by a standard quantitative XPS analysis. The atomic concentrations of the constituents were calculated from the areas of the characteristic photoelectron lines assuming a Shirley type background. All high-resolution spectra were recorded using analyzer pass energy of 20 eV and analyzed after the subtraction of a Shirley background. Casa XPS (Casa Software Ltd., UK, Version 2.3.26PR1.0) software was used for the deconvolution of photoelectron peaks, considering GL (30–40) line shapes.

The surface morphology was investigated using scanning electron microscopy (SEM, VEGA3 SBU, Tescan, Brno-Kohoutovice, Czech Republic) and the elemental composition was determined through energy-dispersive X-ray spectroscopy (EDX, Quantax EDS, Bruker, Karlsruhe, Germany) at room temperature. Small samples were coated with a 10 nm gold layer using a Leica EM ACE200 (LEICA, Wetzlar, Germany).

### 2.5. Cell Culture and MTT Assay

The HaCaT immortalized human keratinocyte cells supplied by CLS Cell Lines Service (Eppelheim, Germany) were cultured in 96-well plates at a density of 9000 cells per well. The cells were allowed to adhere and attach for 24 h prior to treatment. Cell viability was assessed using the MTT (3-(4,5-dimethylthiazol-2-yl)-2,5-diphenyltetrazolium bromide) assay (MTT, Sigma-Aldrich, Saint Louis, MO, SUA) [38,39]. After the 24-h treatment period, the MTT solution (0.5 mg/mL) was added to each well and the plates were incubated at 37 °C for 1 h to allow for the formation of formazan crystals. The media was then removed, and the crystals were dissolved using dimethyl sulfoxide (DMSO, Sigma Aldrich, Saint Louis, MO, USA). Absorbance was measured at 570 nm using a Tecan Infinite 200 Pro reader plate reader. The experiments were conducted on microplates with three identical parallel wells. The results were expressed as percentage of cell viability relative to the untreated control cells, which were assigned a value of 100% viability. The data were presented graphically as mean ± SD.

### 2.6. Antibacterial Activity

The antibacterial activity of the samples was assessed on the Gram-positive bacterium *Staphylococcus aureus* (ATCC 1109R) and the Gram-negative bacterium *Escherichia coli* (ATCC 25922). For each sample, a quantity of 30 mg was mixed with the bacterial suspension in liquid culture (10^4^ cells/mL). Then, 25 μL of the culture was plated on solid medium (Mueller-Hinton agar) and incubated overnight at 37 °C (the cells were plated in triplicate). Negative control (NC) was performed in the same way but without the samples. The Colony Forming Units (CFUs) were counted the following day. All experiment were conducted in triplicate. The data were presented graphically as mean ± SD.

## 3. Results and Discussion

### 3.1. Characterization of the as-Prepared Samples

**Figure 1:**
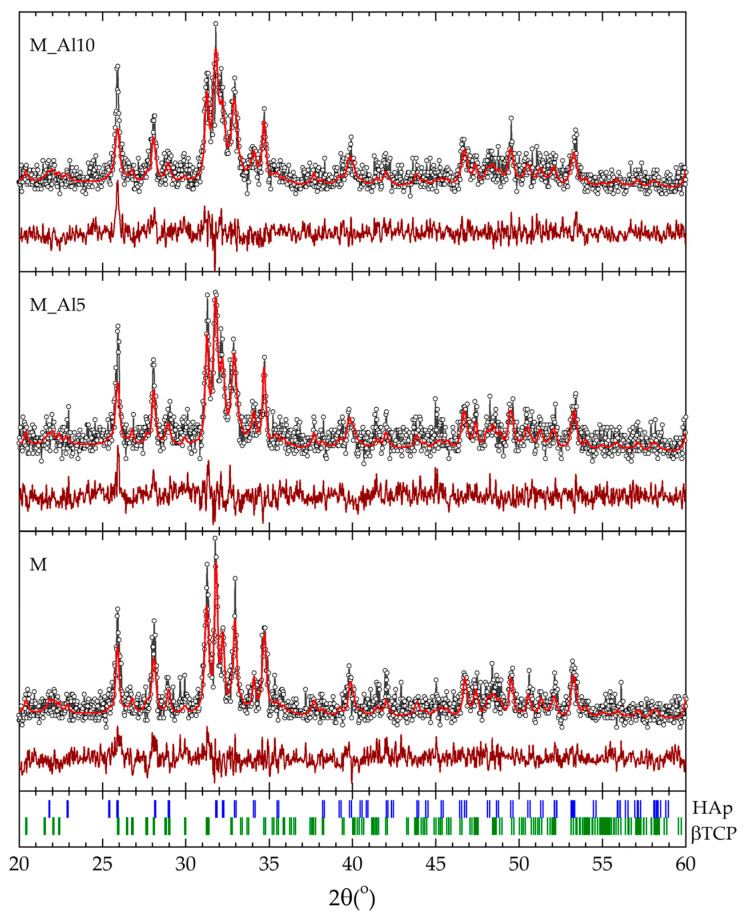
Rietveld refinement of the XRD patterns for the as-prepared samples (the experimental data points are shown as dots, and the calculated profile and difference curve are shown as solid lines) with the respective Bragg positions of the HAp and β-TCP crystal structures (bottom).

The crystalline structure of the as-prepared samples was investigated by X-ray diffraction. XRD patterns indicate the presence of HAp and β-TCP in the as-prepared samples. To estimate the phase distribution, the data collected within the 2θ range of 20° to 60° was analyzed using the Rietveld refinement method. The calculated profiles are shown in Figure 1 together with the experimental data and the results are presented in Table 1. All diffraction maxima are attributed to the hexagonal HAp *P6*_3_/*m* and rhombohedral β-TCP *R3c* structures. The Al-doped samples show a significantly higher content of HAp, while the lattice parameters seem to be unaffected by the Al content and in very good agreement with the values reported in literature for the two phases [7,8,26,40,41,42,43,44].

**Figure 2:**
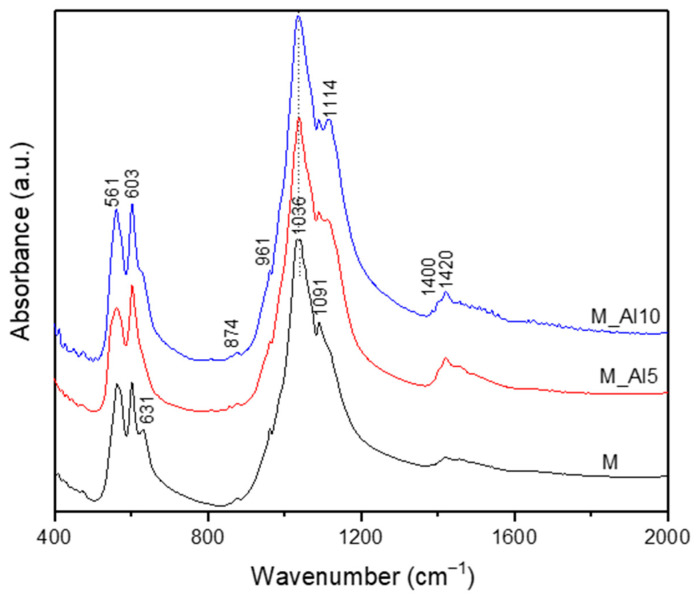
The FTIR spectra of the as-prepared samples.

The FTIR spectra (Figure 2) confirmed the presence of functional groups characteristic for both HAp and β-TCP. The doublet at 560–605 cm^−1^ is assigned to the triple degenerate asymmetric P-O bending mode (ν_4_) from PO_4_^3−^ orthophosphate units from HAp [8,45,46,47,48,49]. Their separation reflects the crystalline nature of the samples [8]. Small peaks are observed around 631 and 3572 cm^−1^ corresponding to the bending, respectively stretching vibration of structural hydroxyl (OH^−^) groups. These bands indicate, according to the literature, the formation of the HAp structure with low crystallinity [45,46,47,48]. The peak at 961 cm^−1^ is characteristic to symmetric stretching (ν_1_) vibration of P-O from PO_4_^3−^ units in hydroxyapatite [8,47,50]. The strong peak at 1036 cm^−1^ and higher-wavenumber inflections at 1091 cm^−1^ are assigned to P-O asymmetric stretching vibration (ν_3_) [51,52]. Since literature indicates for pure HAp a strong peak at 1300 cm^−1^ in the FTIR spectra and for the b-TCP phase a broader peak at 1040 cm^−1^, we assume a contribution of both structural phases at the peak observed at 1036 cm^−1^ [47,48,49,50,51]. In the FTIR spectra of Al-doped samples, a peak at 1114 cm^−1^ is present because of the P-O asymmetric stretching vibration (ν_3_) in b-TCP [53,54]. This suggests that the aluminium ions effectively modify the structure and crystallinity of the samples. The FTIR spectra of samples present additional small bands at 874 and 1400–1420 cm^−1^ assigned to the asymmetric stretching (ν_3_) and bending (ν_2_) vibration mode of CO_3_^2−^ [47,51]. These bands are stronger in Al-containing samples and are characteristic to the b-TCP phase [26,53]. Overall, the spectra corroborate a mixed HAp/β-TCP composition with carbonate substitution and Al-dependent spectral shifts.

In addition to FTIR spectroscopy, the Raman spectroscopy is a powerful tool for elucidating the local structure of glasses, with both techniques providing complementary insights. It is well established that a strong band in the Raman spectrum of a given compound typically corresponds to a weak band in its FTIR spectrum, and vice versa [55,56]. This complementary relationship arises due to the differing selection rules governing vibrational transitions in these spectroscopic methods. While FTIR spectroscopy primarily detects dipole moment changes associated with vibrational modes, the Raman spectroscopy is sensitive to polarizability variations within molecular bonds. Thus, the combined use of the FTIR and Raman spectroscopic techniques enables a more comprehensive characterization of glass structure, facilitating a deeper understanding of network connectivity, bond environments, and structural transformations.

**Figure 3:**
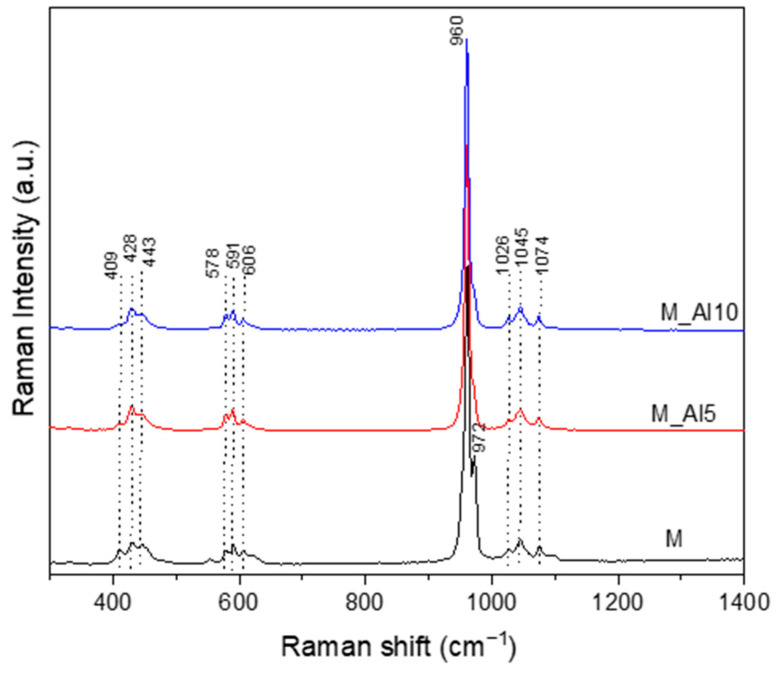
Raman spectra of as-prepared samples.

The Raman spectra, illustrated in Figure 3, present an intense maximum at 960 cm^−1^ corresponding to the ν_1_ vibration of PO_4_^3−^, the symmetric stretching of P–O bonds. This band is reported in the literature at 964 cm^−1^ for commercial HAp and 961 cm^−1^ for bone, with small shifts for β -type carbonated hydroxyapatite [46,57]. In biphasic calcium phosphate compositions, the ~960 cm^−1^ band reflect the formation of hydroxyapatite phase [58]. The Raman spectra of sample M present a sharp band at 972 cm^−1^ related to ν_1_ symmetric stretching of PO_4_^3−^ characteristic of β-tricalcium phosphate [46]. A visible broadening of this band with Al-ion addition indicates a lattice rearrangement that suppresses the β-TCP phase. In the 400–450 cm^−1^ region, three weak peaks were detected. The Raman band at ~409 cm^−1^, most clearly resolved in sample M, is assigned to the ν_2_ (O–P–O) bending mode of the phosphate group (PO_4_^3−^), characteristic of β-tricalcium phosphate (β-TCP) and detectable in dicalcium phosphate phases [46,58]. Bands centred at 428 and 443 cm^−1^ were attributed to the bending (ν_2_) vibrational mode of P-O bonds from PO_4_^3−^ units in both HAp and β-TCP phases [58,59,60,61]. The asymmetric bending vibration, ν_4_ of P-O bonds generates Raman bands in the 550–650 cm^−1^ domain [59]. In the region around 100–1100 cm^−1^, three bands assigned to the asymmetric stretching vibration, ν_3_ of P–O bond, is identified [57,58,59,60,61].

Because the β-TCP unit cell contains 42 PO_4_^3−^ tetrahedra, whereas stoichiometric hydroxyapatite (HAp) contains only six, the spread of the split PO_4_^3−^ band frequencies in the Raman spectrum is markedly broader in β-TCP [58,62]. Moreover, the separation between bands of the doubly degenerate ν_2_ and triply degenerate ν_4_ modes is substantially smaller in β-TCP (~55 cm^−1^) than in HAp (~120 cm^−1^) [58]. These observations indicate an Al-driven lattice rearrangement that destabilizes β-TCP and shifts the phase assemblage toward an HAp-dominant structure.

**Figure 4:**
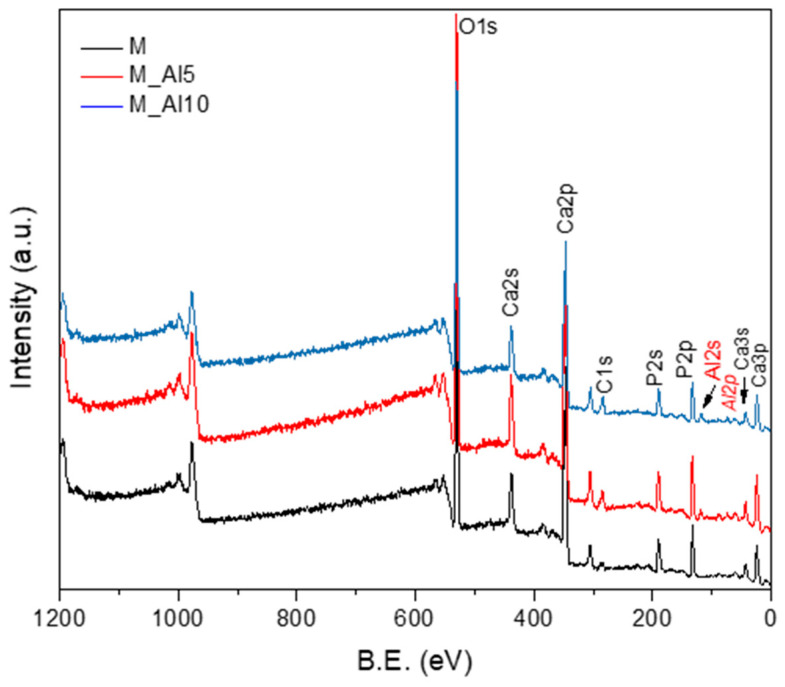
XPS survey spectra of as-prepared samples.

The X-ray photoelectron spectroscopy (XPS) is one of the most widely used techniques of surface analysis used to determine, from binding energy (BE) of ejected electrons, the elemental composition of materials to a depth of up to 2.5 nm from a surface. Even though the surface may not be representative of the bulk material due to an eventual contamination by reaction and/or deposition, it is essential to characterize it, since it is this outer surface that first contacts body fluids on implant [63]. The XPS survey spectra of the synthesized samples, shown in Figure 4, provide insights into their surface composition. The survey spectra were analyzed in terms of peak intensities and positions, to identify all constituent elements (O, P, Ca, Al). The elemental surface compositions of the samples, determined from the XPS analysis, are reported in Table 2, reflecting the distribution of surface species. The Ca/P ratio in the analyzed samples are lower than those for pure HAp (1.66) or β-TCP phase (1.5) reflecting the biphasic nature of these samples.

**Figure 5:**
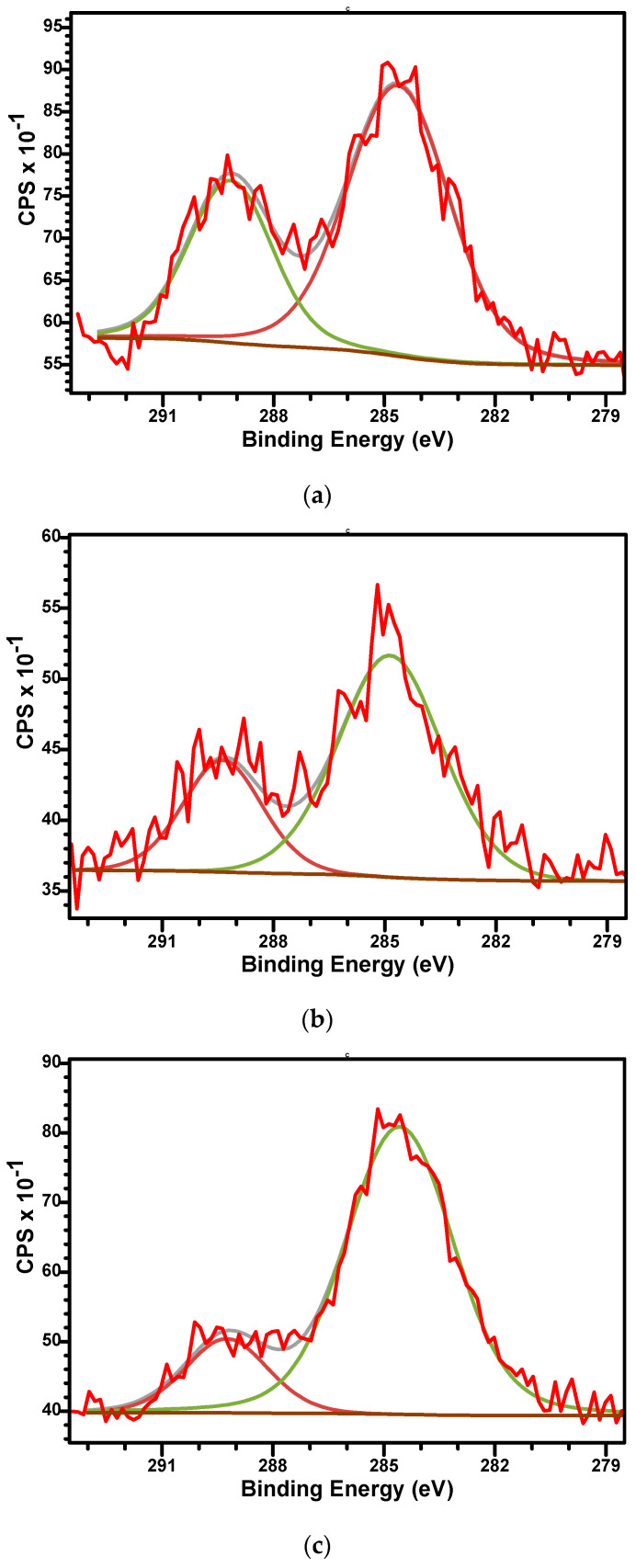
The high-resolution XPS spectra of C1s and curve-fitting results of M (**a**), M_Al5 (**b**), and M_Al10 (**c**) samples.

**Figure 6:**
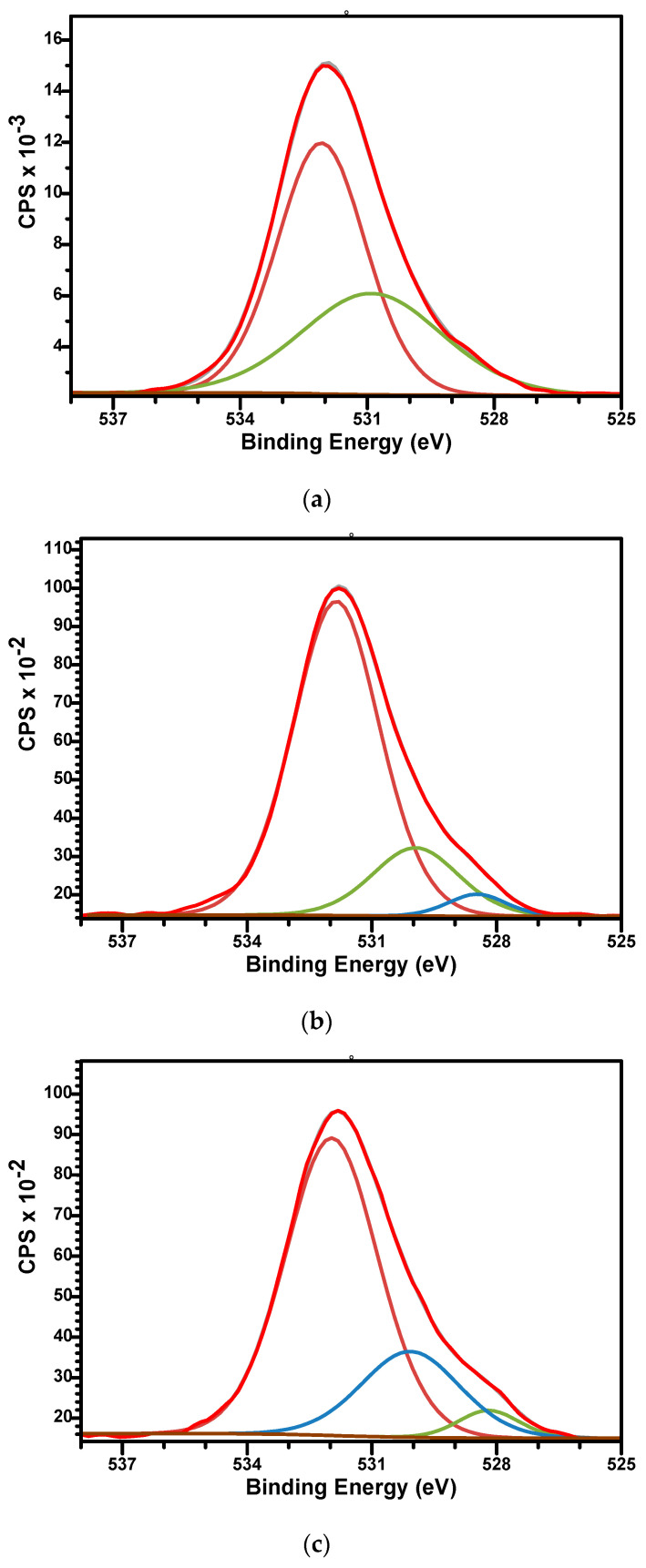
The high-resolution XPS spectra of O1s and curve-fitting results of M (**a**), M_Al5 (**b**), and M_Al10 (**c**) samples.

**Figure 7:**
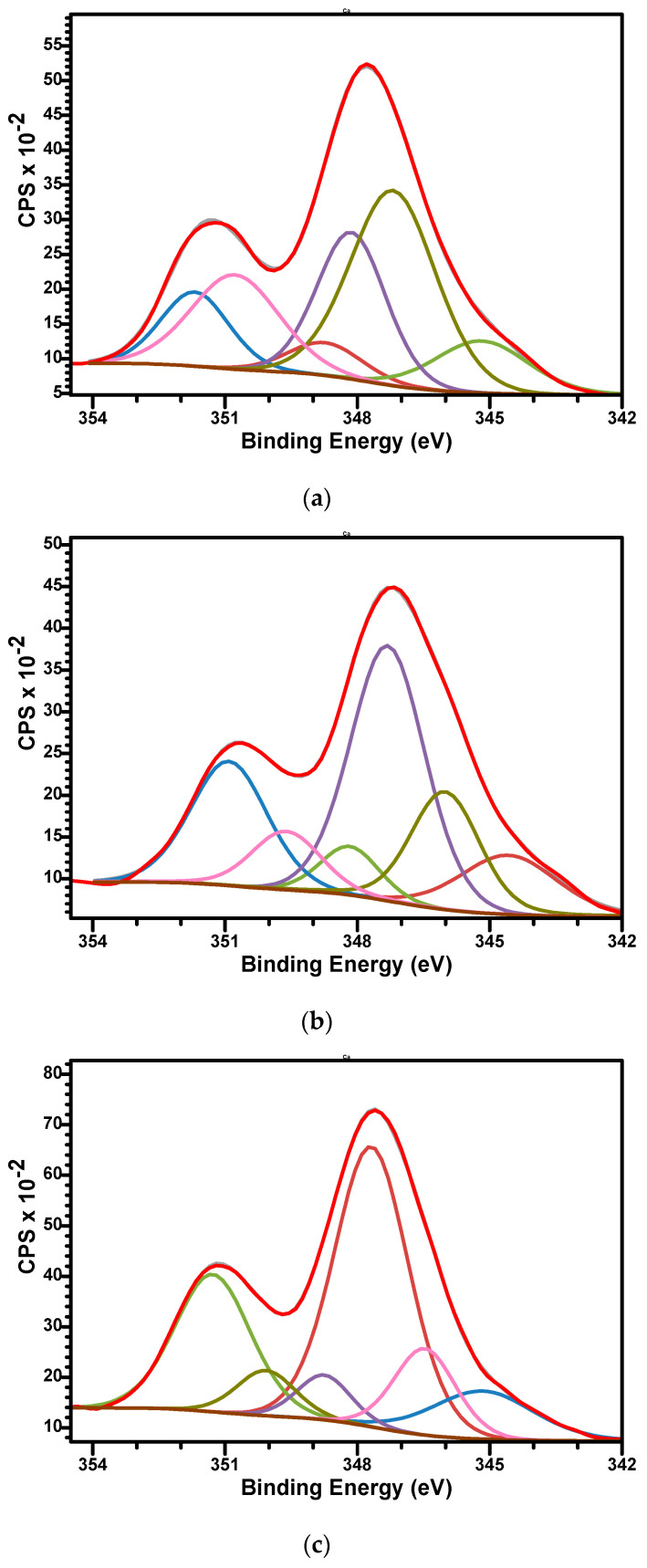
The high-resolution XPS spectra of Ca2p and curve-fitting results of M (**a**), M_Al5 (**b**), and M_Al10 (**c**) samples.

**Figure 8:**
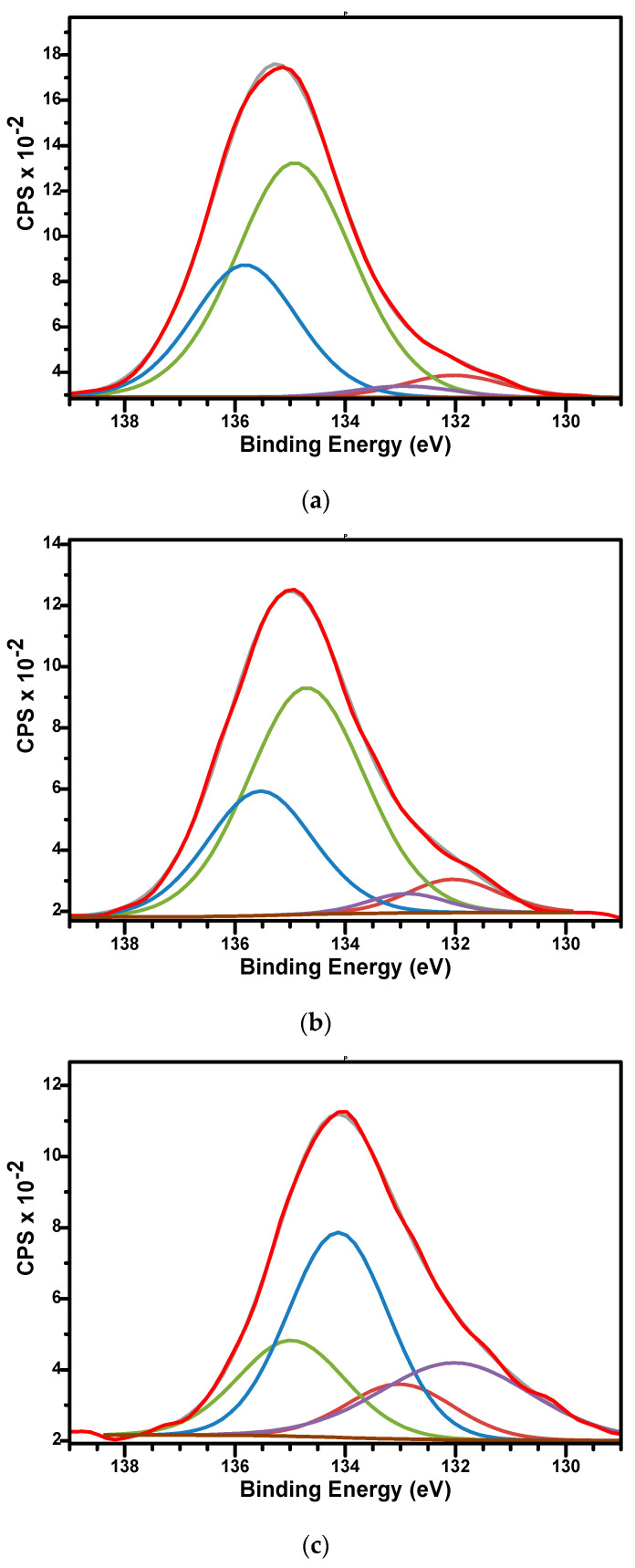
The high-resolution XPS spectra of P2p and curve-fitting results of M (**a**), M_Al5 (**b**), and M_Al10 (**c**) samples.

The high-resolution XPS spectra of C1s, O1s, Ca2p, and P2p for all as-prepared samples are presented in Figure 5, Figure 6, Figure 7 and Figure 8. The high-resolution spectra of C1s (Figure 5) shows two distinct signals in narrow ranges of 284.6–284.8 eV and 289.2–289.4 eV. These energies were associated with the C-C bond (284.6–284.8 eV) and with carboxyl groups O-C=O (289.2–289.4 eV). Second component confirms the presence of carbonate group (CO_3_^2−^) within the material’s structure and, in consequence, the development of β-TCP phase in all samples. The ratio between the areas that cover the C-C and O-C=O contribution in the high-resolution spectra increases with the aluminium ions content increased from 2.06 to 2.6 and 5.34. This suggests that Al^3+^ disadvantages the β-TCP phase, also observed in the XRD analysis.

The high-resolution spectra of O1s for sample M (Figure 6a) can be fitted by two components while the spectra of M_Al5 and M_Al10 request three. For sample M, the signal at BE of 532.08 eV is associated with the P-O bonds while the signal at BE of 530.89 eV is associated with P-O, hydroxyl (OH^−^) groups, and C-O bonds [47]. For Al-containing samples (Figure 6b,c), three peaks were used to fit the signal with BE around 531.8, 530, and 528.5 eV. The first two peaks were associated similar with those of sample M while the lowest signal at 528.5 eV could be assigned to the Al-O bonds [64].

The high-resolution XPS spectra of Ca2p for samples are presented in Figure 7a–c. Three doublets for Ca2p, corresponding each doublet to 2p3/2 and 2p1/2 split, spaced at approximately 3.55 eV and with an area ratio 1:2, were used to fit the signals. The Ca 2p3/2 binding energy values were observed as follows: at 345.18, 347.19, and 348.13 eV (sample M); at 345.15, 346.47, and 347.68 eV (sample M_Al5) and 345.1, 346.02, and 347.32 eV (M_Al10). The binding energies are specific to biphasic calcium phosphate materials, but a specific association of the signal is difficult since the divalent Ca^2+^ occupied two sites in HAp and five sites in β-TCP structures [65]. This peculiarity generates differences in their electronic structures and therefore differences in their atomic binding energies. We attribute the Ca 2p_3_/_2_ component characteristic of CaCO_3_ to the lower BE (~345.1), whereas the higher BEs are consistent with Ca in calcium-phosphate (BCP: HAp/β-TCP) environments.

The high-resolution XPS spectra of P2p for samples are presented in Figure 8a–c. The spectra were fitted using two doublets, each covering peaks of P 2p3/2 and P 2p1/2 contribution, spaced by approximately 0.86 eV and with an area ratio of 0.5. The binding energies for P 2p3/2 signals were 132.04 and 134.7 eV for sample M; 132.03 and 134.8 eV for sample M_Al5; and 132.01 and 134.12 eV for sample M_Al10, respectively. The binding energies around 132 eV for P 2p3/2 signals were specific to orthophosphate PO_4_^3−^ units characteristics for hydroxyapatite while the binding energies around 134.5 eV were characteristics to the metaphosphate (P-O-P bonds) structure [64,66].

**Figure 9:**
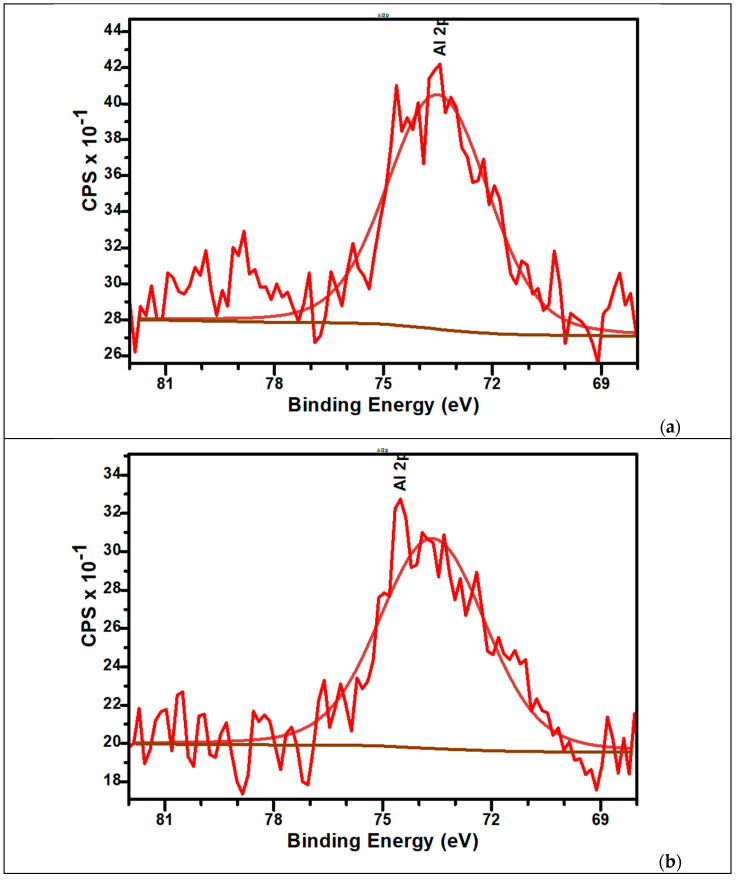
The high-resolution XPS spectra of Al2p and curve-fitting results of M_Al5 (**a**) and M_Al10 (**b**) samples.

The high-resolution XPS spectra of Al2p for samples are presented in Figure 9a,b. The signals present a small shift to lower binding energy at the aluminium content increases in sample. For M_Al5, the Al2p peak is centred at 75.4 eV while for M_Al10 appears at 75 eV. These values are indicators for the presence of Al^3+^ bonds with Ca^2+^ (via oxygen atoms bridges), PO_4_^2−^ and/or PO_4_^3−^.

**Figure 10:**
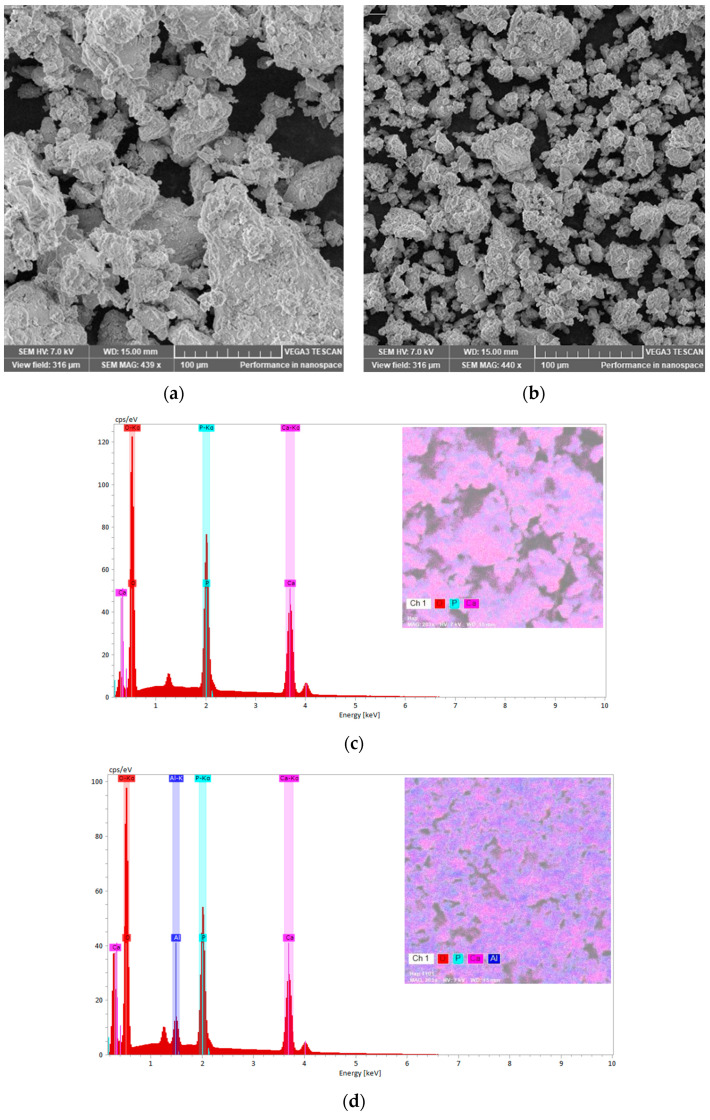
SEM images and EDX spectra of samples M (**a**,**c**) and M_Al10 (**b**,**d**) with elemental maps.

To follow the morphology and the aggregation tendency of the biphasic calcium phosphate sample with the aluminium additions, SEM images were taken (Figure 10a,b). Moreover, EDX spectra were recorded to check the samples composition (Figure 10c,d). The SEM images indicate the distribution of the constitutive atoms in agglomerates, which consist of smaller particles with a wide range size distribution for sample M. Increasing the Al content produces a marked reduction in particle size, consistent with an Al-driven rearrangement toward an HAp-dominated structure. XRD analysis corroborates this trend (Table 1). Raman spectra likewise indicate this evolution. The β-TCP ν_1_(PO_4_) band at 972 cm^−1^ is nearly extinguished upon Al addition, while the HAp ν_1_(PO_4_) band at 960 cm^−1^ becomes dominant. The energy-dispersive X-ray spectroscopy (EDX) confirms the composition, detecting Ca and P consistent with calcium-phosphate phases together with Al. The EDX maps presented in pseudo-colour representation illustrate the elemental distribution across the sample surfaces. For sample M, the map reveals a relatively homogeneous dispersion of Ca and phosphorus P with localized oxygen enrichments. In contrast, the map for the M_Al10 sample indicates a uniform distribution of Al on the surface, with observable agglomeration of calcium and phosphorus. These observations corroborate the findings obtained through the Rietveld refinement analysis.

### 3.2. Characterization of the Composite Samples

**Figure 11:**
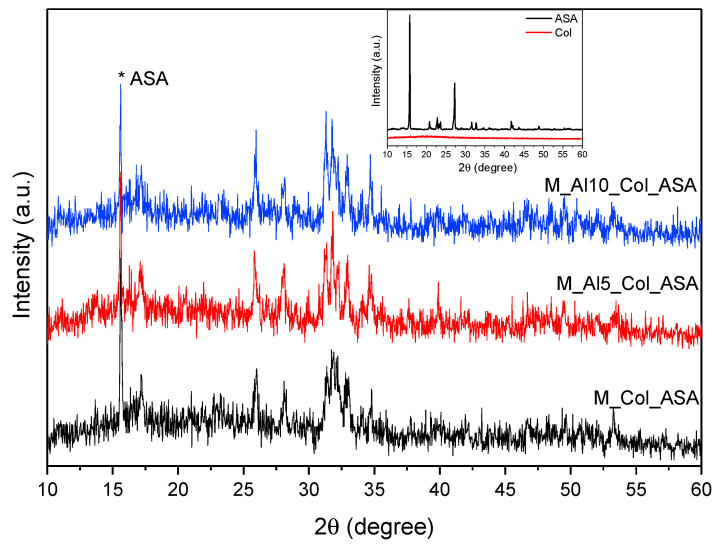
XRD patterns of the composites. The reference spectra for collagen (Col) and acetylsalicylic acid (ASA) are shown in the inset.

Figure 11 compares the XRD patterns of the composite samples with those of pure aspirin (ASA) and collagen (COL). The prominent ASA reflection at 2θ ≈ 15.72° appears in all three composites, indicating successful drug incorporation. In addition to this feature, the composites retain the characteristic reflections of biphasic calcium phosphate (BCP), consistent with preservation of the underlying HAp/β-TCP framework. Because collagen hydrolysate is largely amorphous, XRD is not diagnostic for its incorporation. The composite patterns exhibit a modest attenuation of BCP peak intensities relative to the pristine ceramic, attributable to dilution and partial scattering/absorption by the organic constituents.

**Figure 12:**
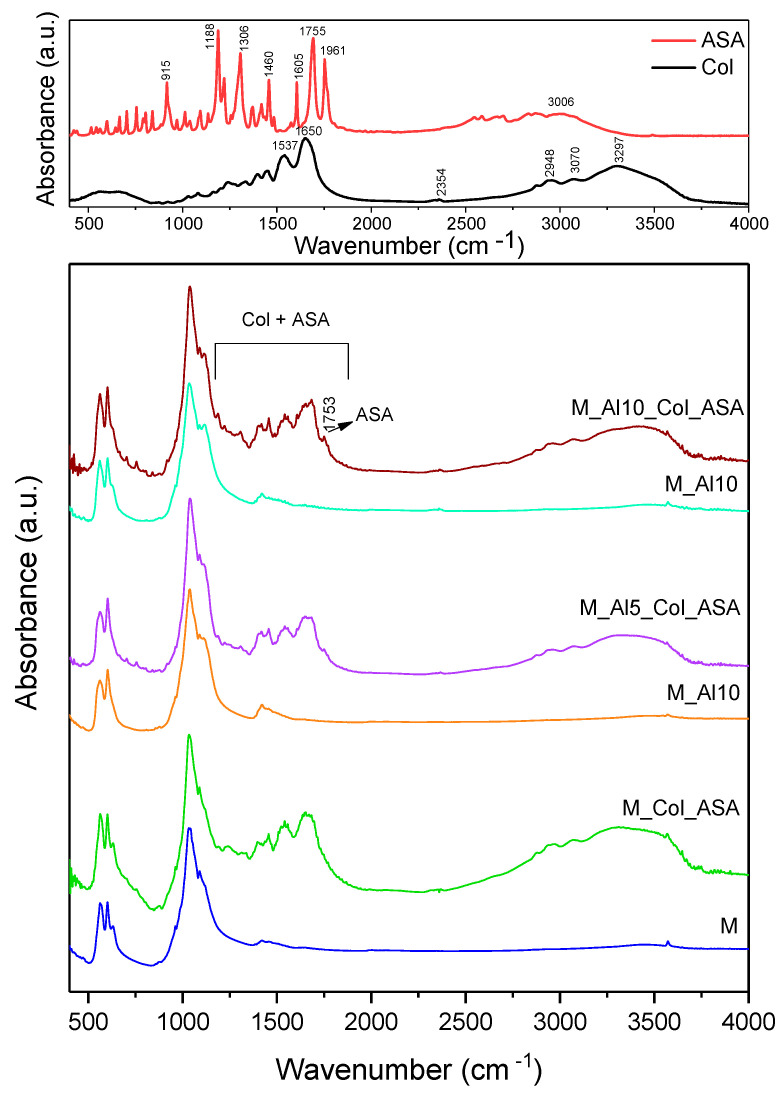
Comparative FTIR spectra of as-prepared samples and composites. The reference spectra for collagen (Col) and acetylsalicylic acid (ASA) are shown in the top.

Figure 12 presents the FTIR spectra of acetylsalicylic acid (ASA), collagen (Col), as prepared and composite samples. The FTIR spectrum of ASA presents in the 500–2000 cm^−1^ domain multiple sharp peaks, very strong at 1755 cm^−1^ associated with ester C=O stretch, strong at 1961 cm^−1^ due to carboxylic-acid C=O stretch (H-bonded), medium at ~1605 and 1460 cm^−1^ assigned to aromatic C=C stretches, strong at 1306 and 1188 cm^−1^ due to C–O stretches from both, ester (–COOR) and carboxylic acid (–COOH) groups [67,68]. The FTIR band at 915 cm^−1^ is commonly assigned to an aromatic C–H out-of-plane bending vibration of the substituted benzene ring [67,68]. Broad bands associated to O–H stretching vibration in the 2500–3300 cm^−1^ spectral range were also observed. The infrared spectrum of collagen shows clear bands at 1650 cm^−1^ (Amide I, C=O stretching vibrations of the peptide bond), 1537 cm^−1^ (Amide II, primarily N–H bending with C–N stretching vibrations), and 1236 cm^−1^ (Amide III, mixed C–N stretching and N–H in-plane bending vibrations) [69,70,71]. A large band was observed at 3297 cm^−1^ as a contribution of Amide A (N–H bond stretching in the collagen triple helix) [72]. The FTIR spectra of the composites appear as superpositions (envelopes) of bands from the as-prepared ceramic, acetylsalicylic acid (ASA), and collagen. In the 1200–1700 cm^−1^ region and near 3300 cm^−1^, all characteristic collagen bands are observed. In addition, a very weak band at 1753 cm^−1^ is discernible for the Al-containing composites, consistent with ASA incorporation and successful composite formation.

**Figure 13:**
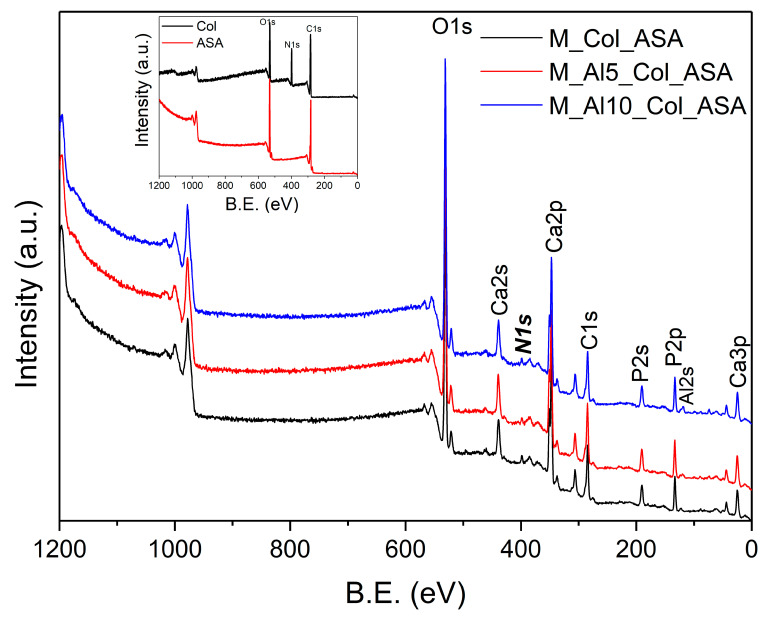
The XPS survey spectra of the composites. The reference spectra for collagen (Col) and acetylsalicylic acid (ASA) are shown in the inset.

The survey XPS spectra (Figure 13) of the composites reproduce the principal features of the as-prepared powders (Figure 4) and display an additional N 1s peak, evidencing ASA loading. Although collagen cannot be uniquely identified by XPS (it contributes primarily C and O), the composites exhibit higher surface C and O atomic percentages than the as-prepared samples (Table 3), indicating enrichment of both ASA and Col at the outermost surface, advantageous for bone-regeneration applications. The Ca/P atomic ratio remains approximately unchanged. Notably, the surface Al^3+^ content decreases in the composites, likely due to coverage/dilution by the organic overlayer.

**Figure 14:**
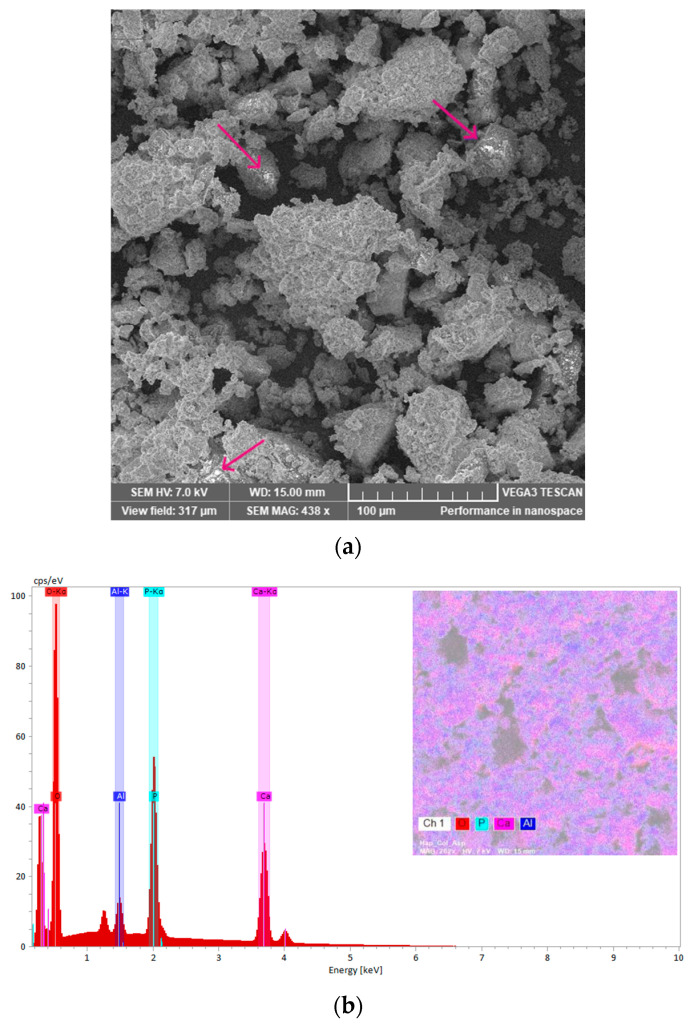
The SEM micrograph of the M_Al10_Col_ASA sample with ASA-rich domains marked by arrows (**a**) and EDX spectra with elemental map (**b**).

Additional morphological information was obtained by scanning electron microscopy (SEM). Figure 14a shows the SEM image of the M_Al10_Col_ASA sample, revealing a multigranular, heterogeneous surface with a broad crystallite-size distribution. Compared with the as-prepared material (Figure 10b), the composite exhibits increased agglomeration, attributable to the incorporation of collagen and ASA. Bright spots, assigned to ASA-rich domains, are also visible (see inset in Figure 14a). Figure 14b presents the energy-dispersive X-ray (EDX) spectrum of the M_Al10_Col_ASA sample; pseudo-colour elemental map illustrates the distribution across the sample surface.

**Figure 15:**
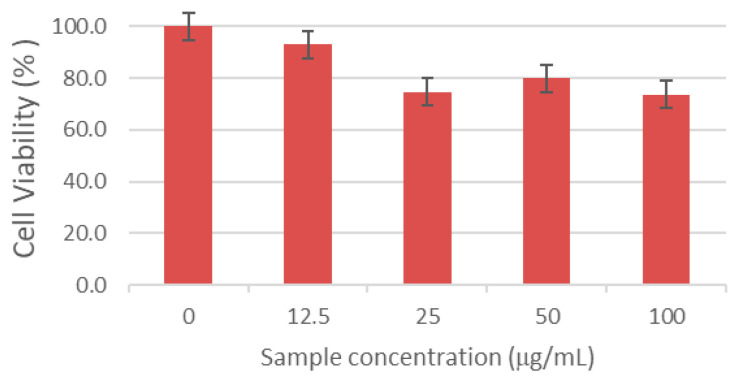
HaCaT cell viability after 24 h exposure to serial dilutions of the M_Al10_Col_ASA composite. The results are presented as the mean ± standard deviation and quantified as percentages of the control representing (100% cell viability).

Implant success is largely determined by cell–biomaterial interactions at the surface. Insufficient adhesion can impair tissue repair and compromise osseointegration. To assess cytocompatibility, serial dilutions of the composites were tested with HaCaT keratinocytes. Although the target tissue of composites is bone, ISO 10993 [73] recommends an initial, standardized cytotoxicity screen to detect extract-mediated effects before lineage-specific assays. HaCaT keratinocytes were used here as a conservative first-pass model because (*i*) implantation in dentoalveolar/craniofacial settings transiently exposes keratinized epithelia to leachable, and (*ii*) keratinocytes are sensitive to shifts in pH/ionic strength and to small-molecule or ionic releases (e.g., ASA; Ca/P/Al species), thus providing an early safety flag under extract conditions. Cell viability after 24 h was quantified by the MTT assay and expressed as a percentage of the untreated control (mean ± SD; control = 100%; Figure 15). All formulations showed viability of at least 70% across the tested dilutions, meeting the ISO 10993-5 criterion for non-cytotoxicity [73]. Notably, the M_AL10_Col_ASA composite at 12.5% yielded the highest viability (>90%), indicating excellent cytocompatibility and underscoring its promise for biomedical applications.

**Figure 16:**
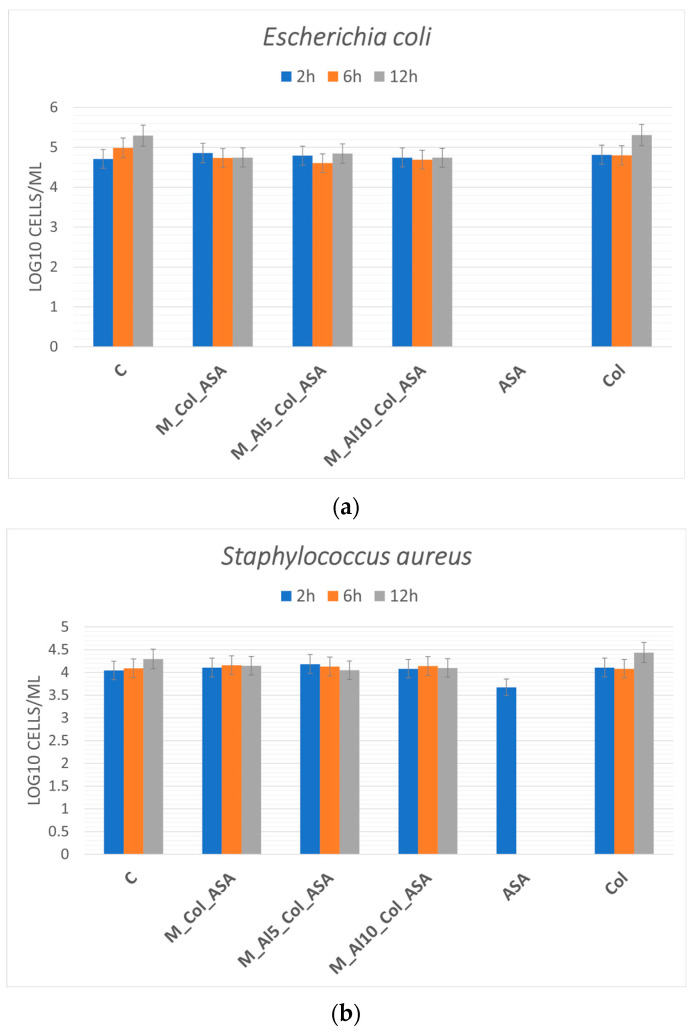
Antibacterial activity of the composites against *Escherichia coli* (**a**) and *Staphylococcus aureus* (**b**) after 2, 6, and 12 h of exposure.

The in vitro antibacterial response of the composites was assessed against *Escherichia coli* (ATCC 25922) and *Staphylococcus aureus* (ATCC 1109R). CFUs were quantified at 2, 6, and 12 h, and results are shown as mean ± standard error (n = 3) in Figure 16a,b. In bone-regenerative biomaterials, acetylsalicylic acid (ASA) can act as a bioactive additive that—via cyclo-oxygenase (COX) inhibition and consequent prostaglandin E_2_ suppression—attenuates inflammation, modulates osteoblast–osteoclast activity, and may provide adjunct antibacterial/biofilm-modulating effects under controlled release [5,32,33,34,35,74]. Within the present variability, the small decreases observed for *E. coli* at 6–12 h did not exceed the associated standard errors and are therefore interpreted as non-significant trends. For *S. aureus*, CFU values were consistently lower than the negative control across time points, indicating a time-dependent bacteriostatic tendency; however, these differences likewise did not reach statistical significance under the current sampling. Notably, even modest bacteriostatic effects are relevant for implantable biomaterials because they curb early bacterial proliferation, allowing the host immune system (and adjunct antibiotics, if used) to clear the residual burden. Accordingly, under the tested conditions the composites exhibit modest, time-dependent bacteriostatic behavior consistent with ASA’s strain- and dose-dependent adjunct activity; larger sample sizes, optimized ASA loading/release, and formal hypothesis testing are warranted to resolve statistical differences.

## 4. Conclusions

This study reports the successful synthesis of Al-modified biphasic calcium phosphate (Al-BCP) ceramics and Al-BCP–collagen–aspirin (Al-BCP–Col–ASA) composites. All materials are crystalline and display signatures of both hydroxyapatite (HAp) and β-tricalcium phosphate (β-TCP). Aluminium incorporation shifts the phase assemblage toward HAp and, in the composites, promotes stronger interfacial retention/adhesion of Col and ASA. Spectroscopic and surface analyses (FTIR, Raman, XPS) and SEM observations consistently support efficient organic loading and uniform surface coverage

Cytocompatibility assays with HaCaT keratinocytes showed more than 70% viability across tested dilutions, meeting ISO 10993-5 criteria for non-cytotoxicity, with the M_AL10_Col_ASA formulation at 12.5% achieving >90% viability. In vitro, preliminary antibacterial tests demonstrated a time-dependent modest bacteriostatic effect against *Staphylococcus aureus*. These results indicate that the composites can suppress early bacterial colonization while maintaining cell viability, thereby supporting conditions favorable for stable implant–bone integration.

Overall, the aluminium-modified new BCP–Col–ASA system is a promising candidate for bone-regeneration applications. Future work should quantify ASA release kinetics and anti-inflammatory efficacy; correlate antibacterial activity with dose and exposure time; and evaluate degradation/resorption under physiological conditions, followed by in vivo testing.

## Figures and Tables

**Table 1:** Rietveld refinement results of relative amounts and lattice parameters for the two identified crystalline phases in the XRD patterns.

Sample	HAp			β-TCP		
	%	a (Å)	c (Å)	%	a (Å)	c (Å)
M	54.8	9.41 (4)	6.88 (2)	45.2	10.33 (6)	37.21 (7)
M_Al5	68.6	9.42 (5)	6.88 (3)	31.4	10.34 (0)	37.18 (5)
M_Al10	68.7	9.42 (2)	6.87 (7)	31.3	10.34 (2)	37.19 (6)

**Table 2:** The elemental composition of as-prepared samples determined from the XPS analysis with an accuracy of ± 0.2 at %.

Sample	Atomic %				Ratio
	Ca	P	O	Al	Ca/P
**M**	25.24	22.90	51.86	-	1.10
**M_Al5**	23.87	21.12	51.87	3.14	1.13
**M_Al10**	22.21	19.59	51.56	6.64	1.13

**Table 3:** The elemental composition of composite samples determined from the XPS analysis with an accuracy of ±0.2 at %.

Sample		Atomic %				Ratio
	Ca	P	O	N	Al	Ca/P
M_Col_ASA	22.63	19.60	55.55	2.22	-	1.15
M_Al5_Col_ASA	21.35	18.57	55.89	1.98	2.26	1.15
M_Al10_Col_ASA	19.93	17.14	55.82	2.05	5.05	1.16

## Data Availability

The original contributions presented in the study are included in the article. Further inquiries can be directed at the corresponding authors.

## References

[1] De Pace R., Molinari S., Mazzoni E., Perale G. (2025). Bone Regeneration: A Review of Current Treatment Strategies. J. Clin. Med..

[2] Georgeanu V.A., Gingu O., Antoniac I.V., Manolea H.O. (2023). Current Options and Future Perspectives on Bone Graft and Biomaterials Substitutes for Bone Repair, from Clinical Needs to Advanced Biomaterials Research. Appl. Sci..

[3] Rodríguez-Merchán E.C., Gómez-Cardero P., Encinas-Ullán C.A. (2021). Management of Bone Loss in Revision Total Knee Arthroplasty: Therapeutic Options and Results. EFORT Open Rev..

[4] Su N., Villicana C., Yang F. (2022). Immunomodulatory Strategies for Bone Regeneration: A Review from the Perspective of Disease Types. Biomaterials.

[5] Zhang H., Qiao W., Liu Y., Yao X., Zhai Y., Du L. (2025). Addressing the Challenges of Infectious Bone Defects: A Review of Recent Advances in Bifunctional Biomaterials. J. Nanobiotechnol..

[6] Hao X., Jiang B., Wu J., Xiang D., Xiong Z., Li C., Li Z., He S., Tu C., Li Z. (2024). Nanomaterials for Bone Metastasis. J. Control. Release.

[7] França R., Samani T.D., Bayade G., Yahia L., Sacher E. (2014). Nanoscale Surface Characterization of Biphasic Calcium Phosphate, with Comparisons to Calcium Hydroxyapatite and β-Tricalcium Phosphate Bioceramics. J. Colloid Interface Sci..

[8] Kolmas J., Romaniuk P., Predoi D., Drobniewska A., Burdan K., Kołodziejska B. (2025). Magnesium Ion Substitution in Various Calcium Phosphates: A Way towards Bone Regeneration. Ceram. Int..

[9] Tanvir M.A.H., Khaleque M.A., Kim G.-H., Yoo W.-Y., Kim Y.-Y. (2024). The Role of Bioceramics for Bone Regeneration: History, Mechanisms, and Future Perspectives. Biomimetics.

[10] Xiao D., Zhang J., Zhang C., Barbieri D., Yuan H., Moroni L., Feng G. (2020). The Role of Calcium Phosphate Surface Structure in Osteogenesis and the Mechanisms Involved. Acta Biomater..

[11] Zhang Y., Shu T., Wang S., Liu Z., Cheng Y., Li A., Pei D. (2022). The Osteoinductivity of Calcium Phosphate-Based Biomaterials: A Tight Interaction with Bone Healing. Front. Bioeng. Biotechnol..

[12] Lu H., Zhou Y., Ma Y., Xiao L., Ji W., Zhang Y., Wang X. (2021). Current Application of Beta-Tricalcium Phosphate in Bone Repair and Its Mechanism to Regulate Osteogenesis. Front. Mater..

[13] Fiume E., Magnaterra G., Rahdar A., Verné E., Baino F. (2021). Hydroxyapatite for Biomedical Applications: A Short Overview. Ceramics.

[14] Dorozhkin S.V. (2022). Calcium Orthophosphate (CaPO_4_)-Based Bioceramics: Preparation, Properties, and Applications. Coatings.

[15] Gao C., Peng S., Feng P., Shuai C. (2017). Bone Biomaterials and Interactions with Stem Cells. Bone Res..

[16] Taherimehr M., Bagheri R., Taherimehr M. (2021). In-Vitro Evaluation of Thermoplastic Starch/Beta-Tricalcium Phosphate Nano-Biocomposite in Bone Tissue Engineering. Ceram. Int..

[17] Hou X., Zhang L., Zhou Z., Luo X., Wang T., Zhao X., Lu B., Chen F., Zheng L. (2022). Calcium Phosphate-Based Biomaterials for Bone Repair. J. Funct. Biomater..

[18] Pandit A., Indurkar A., Locs J., Haugen H.J., Loca D. (2024). Calcium Phosphates: A Key to Next--Generation In Vitro Bone Modeling. Adv. Healthc. Mater..

[19] Liang X., Yuan S., Zhang N., Xiao Z., Guo D., Zhang C., Lu W., Xian G., Zhang L., Xie D. (2025). Modulating the Crystallinity of Biphasic Calcium Phosphate Composites Balances Surface and Ionic Cues to Enhance Osteogenesis via Integrin-Mediated Cytoskeletal Signaling. Mater. Today Bio.

[20] Liu W., Cheong N., He Z., Zhang T. (2025). Application of Hydroxyapatite Composites in Bone Tissue Engineering: A Review. J. Funct. Biomater..

[21] Chacon E.L., Bertolo M.R.V., de Guzzi Plepis A.M., Martins V.C.A., dos Santos G.R., Pinto C.A.L., Pelegrine A.A., Teixeira M.L., Buchaim D.V., Nazari F.M. (2023). Collagen–Chitosan–Hydroxyapatite Composite Scaffolds for Bone Repair in Ovariectomized Rats. Sci. Rep..

[22] Vaiani L., Boccaccio A., Uva A.E., Palumbo G., Piccininni A., Guglielmi P., Cantore S., Santacroce L., Charitos I.A., Ballini A. (2023). Ceramic Materials for Biomedical Applications: An Overview on Properties and Fabrication Processes. J. Funct. Biomater..

[23] Fahami A., Nasiri-Tabrizi B., Beall G.W., Basirun W.J. (2017). Structural Insights of Mechanically Induced Aluminum-Doped Hydroxyapatite Nanoparticles by Rietveld Refinement. Chin. J. Chem. Eng..

[24] Kolekar T.V., Thorat N.D., Yadav H.M., Magalad V.T., Shinde M.A., Bandgar S.S., Kim J.H., Agawane G.L. (2016). Nanocrystalline Hydroxyapatite Doped with Aluminium: A Potential Carrier for Biomedical Applications. Ceram. Int..

[25] Wang M., Wang L., Shi C., Sun T., Zeng Y., Zhu Y. (2016). The Crystal Structure and Chemical State of Aluminum-Doped Hydroxyapatite by Experimental and First Principles Calculation Studies. Phys. Chem. Chem. Phys..

[26] Goldberg M.A., Protsenko P.V., Smirnov V.V., Antonova O.S., Smirnov S.V., Konovalov A.A., Vorckachev K.G., Kudryavtsev E.A., Barinov S.M., Komlev V.S. (2020). The Enhancement of Hydroxyapatite Thermal Stability by Al Doping. J. Mater. Res. Technol..

[27] Nair A.K., Gautieri A., Chang S.-W., Buehler M.J. (2013). Molecular Mechanics of Mineralized Collagen Fibrils in Bone. Nat. Commun..

[28] Kikuchi M., Itoh S., Ichinose S., Shinomiya K., Tanaka J. (2001). Self-Organization Mechanism in a Bone-like Hydroxyapatite/Collagen Nanocomposite Synthesized in Vitro and Its Biological Reaction in Vivo. Biomaterials.

[29] Pek Y.S., Gao S., Arshad M.S.M., Leck K.-J., Ying J.Y. (2008). Porous Collagen-Apatite Nanocomposite Foams as Bone Regeneration Scaffolds. Biomaterials.

[30] Kołodziejska B., Kaflak A., Kolmas J. (2020). Biologically Inspired Collagen/Apatite Composite Biomaterials for Potential Use in Bone Tissue Regeneration—A Review. Materials.

[31] Tamimi F., Kumarasami B., Doillon C., Gbureck U., Nihouannen D.L., Cabarcos E.L., Barralet J.E. (2008). Brushite–Collagen Composites for Bone Regeneration. Acta Biomater..

[32] Ratchford S.M., Lavin K.M., Perkins R.K., Jemiolo B., Trappe S.W., Trappe T.A. (2017). Aspirin as a COX Inhibitor and Anti-Inflammatory Drug in Human Skeletal Muscle. J. Appl. Physiol..

[33] Di Bella S., Luzzati R., Principe L., Zerbato V., Meroni E., Giuffrè M., Crocè L.S., Merlo M., Perotto M., Dolso E. (2022). Aspirin and Infection: A Narrative Review. Biomedicines.

[34] Li S., Xiaowen Y., Yang Y., Liu L., Sun Y., Liu Y., Yin L., Chen Z. (2023). Osteogenic and Anti-Inflammatory Effect of the Multifunctional Bionic Hydrogel Scaffold Loaded with Aspirin and Nano-Hydroxyapatite. Front. Bioeng. Biotechnol..

[35] Zhao Y., Cheng C., Wang X., Yuan Z., Sun B., EL-Newehy M., Abdulhameed M.M., Fang B., Mo X. (2024). Aspirin-Loaded Anti-Inflammatory ZnO-SiO2 Aerogel Scaffolds for Bone Regeneration. ACS Appl. Mater. Interfaces.

[36] Rodríguez-Carvajal J. (1993). Recent Advances in Magnetic Structure Determination by Neutron Powder Diffraction. Phys. B Phys. Condens. Matter.

[37] Rietveld H.M. (1969). A Profile Refinement Method for Nuclear and Magnetic Structures. J. Appl. Crystallogr..

[38] Mosmann T. (1983). Rapid Colorimetric Assay for Cellular Growth and Survival: Application to Proliferation and Cytotoxicity Assays. J. Immunol. Methods.

[39] Denizot F., Lang R. (1986). Rapid Colorimetric Assay for Cell Growth and Survival. J. Immunol. Methods.

[40] Truite C.V.R., Noronha J.N.G., Prado G.C., Santos L.N., Palácios R.S., Do Nascimento A., Volnistem E.A., Da Silva Crozatti T.T., Francisco C.P., Sato F. (2022). Bioperformance Studies of Biphasic Calcium Phosphate Scaffolds Extracted from Fish Bones Impregnated with Free Curcumin and Complexed with β-Cyclodextrin in Bone Regeneration. Biomolecules.

[41] Bohner M., Santoni B.L.G., Döbelin N. (2020). β-Tricalcium Phosphate for Bone Substitution: Synthesis and Properties. Acta Biomater..

[42] Xidaki D., Agrafioti P., Diomatari D., Kaminari A., Tsalavoutas-Psarras E., Alexiou P., Psycharis V., Tsilibary E., Silvestros S., Sagnou M. (2018). Synthesis of Hydroxyapatite, β-Tricalcium Phosphate and Biphasic Calcium Phosphate Particles to Act as Local Delivery Carriers of Curcumin: Loading, Release and In Vitro Studies. Materials.

[43] Yang A., Huang H., Li J., Yang L., Li S., Chang D., Bai Z., Duan G., Guo T., Weng J. (2023). Regulating the Multifactor during Wet Chemical Synthesis to Obtain Calcium Phosphate Powders with Controllable Phase Purity for Bone Repair. Ceram. Int..

[44] Kim D.-H., Hwang K.-H., Lee J.D., Park H.-C., Yoon S.-Y. (2015). Long and Short-Range Order Structural Analysis of In-Situ Formed Biphasic Calcium Phosphates. Biomater. Res..

[45] Ciobanu C.S., Predoi D., Iconaru S.L., Rokosz K., Raaen S., Negrila C.C., Ghegoiu L., Bleotu C., Predoi M.V. (2025). Chrome Doped Hydroxyapatite Enriched with Amoxicillin Layers for Biomedical Applications. Coatings.

[46] Antonakos A., Liarokapis E., Leventouri T. (2007). Micro-Raman and FTIR Studies of Synthetic and Natural Apatites. Biomaterials.

[47] Predoi D., Iconaru S.L., Ciobanu S.C., Predoi S.-A., Buton N., Megier C., Beuran M. (2021). Development of Iron-Doped Hydroxyapatite Coatings. Coatings.

[48] Lebedev V.N., Kharovskaya M.I., Lazoryak B.I., Solovieva A.O., Fadeeva I.V., Amirov A.A., Koliushenkov M.A., Orudzhev F.F., Baryshnikova O.V., Yankova V.G. (2024). Strontium and Copper Co-Doped Multifunctional Calcium Phosphates: Biomimetic and Antibacterial Materials for Bone Implants. Biomimetics.

[49] Liu Q., Matinlinna J.P., Chen Z., Ning C., Ni G., Pan H., Darvell B.W. (2015). Effect of Thermal Treatment on Carbonated Hydroxyapatite: Morphology, Composition, Crystal Characteristics and Solubility. Ceram. Int..

[50] Cimpeanu C., Predoi D., Ciobanu C.S., Iconaru S.L., Rokosz K., Predoi M.V., Raaen S., Badea M.L. (2024). Development of Novel Biocomposites with Antimicrobial-Activity-Based Magnesium-Doped Hydroxyapatite with Amoxicillin. Antibiotics.

[51] Rehman I., Bonfield W. (1997). Characterization of Hydroxyapatite and Carbonated Apatite by Photo Acoustic FTIR Spectroscopy. J. Mat. Sci. Mater. Med..

[52] Rey C., Shimizu M., Collins B., Glimcher M.J. (1991). Resolution-Enhanced Fourier Transform Infrared Spectroscopy Study of the Environment of Phosphate Ion in the Early Deposits of a Solid Phase of Calcium Phosphate in Bone and Enamel and Their Evolution with Age: 2. Investigations in the n_3_ PO_4_ Domain. Calcif. Tissue Int..

[53] Fleet M.E. (2013). The Carbonate Ion in Hydroxyapatite Recent X-Ray and Infrared Results. Front. Biosci..

[54] Wang L., Nancollas G.H. (2008). Calcium Orthophosphates: Crystallization and Dissolution. Chem. Rev..

[55] Neuville D.R., De Ligny D., Henderson G.S. (2014). Advances in Raman Spectroscopy Applied to Earth and Material Sciences. Rev. Mineral. Geochem.

[56] Hadjiivanov K.I., Panayotov D.A., Mihaylov M.Y., Ivanova E.Z., Chakarova K.K., Andonova S.M., Drenchev N.L. (2021). Power of Infrared and Raman Spectroscopies to Characterize Metal-Organic Frameworks and Investigate Their Interaction with Guest Molecules. Chem. Rev..

[57] Jillavenkatesa A., Condrate R.A. (1998). The Infrared and Raman Spectra of β-and α-Tricalcium Phosphate (Ca_3_(PO_4_)_2_). Spectrosc. Lett..

[58] Cuscó R., Guitián F., de Aza S., Artús L. (1998). Differentiation between Hydroxyapatite and β-Tricalcium Phosphate by Means of μ-Raman Spectroscopy. J. Eur. Ceram. Soc..

[59] Monção M.M., Barreto I.C., Miguel F.B., De Oliveira L.F.C., Carrodeguas R.G., De Araújo R.P.C. (2022). Raman Spectroscopy Analysis of Wollastonite/Tricalcium Phosphate Glass-Ceramics after Implantation in Critical Bone Defect in Rats. Mater. Sci. Appl..

[60] Sauer G.R., Zunic W.B., Durig J.R., Wuthier R.E. (1994). Fourier Transform Raman Spectroscopy of Synthetic and Biological Calcium Phosphates. Calcif. Tissue Int..

[61] Unal M., Ahmed R., Mahadevan-Jansen A., Nyman J.S. (2021). Compositional Assessment of Bone by Raman Spectroscopy. Analyst.

[62] Sadovnikova M.A., Murzakhanov F.F., Fadeeva I.V., Forysenkova A.A., Deyneko D.V., Mamin G.V., Gafurov M.R. (2022). Study of Tricalcium Phosphate Ceramics Doped with Gadolinium Ions with Various EPR Techniques. Ceramics.

[63] Kloprogge J.T. (2025). X-Ray Photoelectron Spectroscopy (XPS) Study of Layered Double Hydroxides with Different Exchangeable Anions. Appl. Sci..

[64] Barrère F., Lebugle A., Van Blitterswijk C.A., De Groot K., Layrolle P., Rey C. (2003). Calcium Phosphate Interactions with Titanium Oxide and Alumina Substrates: An XPS Study. J. Mat. Sci. Mater. Med..

[65] López E.O., Bernardo P.L., Checca N.R., Rossi A.L., Mello A., Ellis D.E., Rossi A.M., Terra J. (2022). Hydroxyapatite and Lead-Substituted Hydroxyapatite near-Surface Structures: Novel Modelling of Photoemission Lines from X-Ray Photoelectron Spectra. App. Surf. Sci..

[66] Sinulingga K., Sirait M., Siregar N., Abdullah H. (2021). Synthesis and Characterizations of Natural Limestone-Derived Nano-Hydroxyapatite (HAp): A Comparison Study of Different Metals Doped HAps on Antibacterial Activity. RSC Adv..

[67] Boczar M., Wójcik M.J., Szczeponek K., Jamróz D., Zięba A., Kawałek B. (2003). Theoretical Modeling of Infrared Spectra of Aspirin and Its Deuterated Derivative. Chem. Phys..

[68] Ye Y., Tang G., Han Y., Culnane L.F., Zhao J., Zhang Y. (2016). DFT Studies on the Vibrational and Electronic Spectra of Acetylsalicylic Acid. Opt. Spectrosc..

[69] Payne K.J., Veis A. (1988). Fourier Transform IR Spectroscopy of Collagen and Gelatin Solutions: Deconvolution of the Amide I Band for Conformational Studies. Biopolymers.

[70] Stani C., Vaccari L., Mitri E., Birarda G. (2020). FTIR Investigation of the Secondary Structure of Type I Collagen: New Insight into the Amide III Band. Spectrochim. Acta A Mol. Biomol. Spectrosc..

[71] Martínez Cortizas A., López-Costas O. (2020). Linking Structural and Compositional Changes in Archaeological Human Bone Collagen: An FTIR-ATR Approach. Sci. Rep..

[72] Júnior Z.S.S., Botta S.B., Ana P.A., França C.M., Fernandes K.P.S., Mesquita-Ferrari R.A., Deana A., Bussadori S.K. (2015). Effect of Papain-Based Gel on Type I Collagen—Spectroscopy Applied for Microstructural Analysis. Sci. Rep..

[73] (2009). Biological Evaluation of Medical Devices—Part 5: Tests for In Vitro Cytotoxicity.

[74] Chang Y., Kong K., Tong Z., Qiao H., Hu Y., Xia R., Zhang J., Zhai Z., Li H. (2023). Aspirin Prevents Estrogen Deficiency-Induced Bone Loss by Inhibiting Osteoclastogenesis and Promoting Osteogenesis. J. Orthop. Surg. Res..

